# EHMN2026^®^T: A License-Aware AI-QSP Integration Framework Linking EHMN2026^®^ with TRANSFAC^®^, TRANSPATH^®^ and HumanPSD™ for Diagnostic-Metabolite Interpretation

**DOI:** 10.3390/metabo16070469

**Published:** 2026-07-04

**Authors:** Igor Goryanin, Leonid Slovianov, Irina V. Goryanin, Alexander Kel

**Affiliations:** 1 School of Informatics, University of Edinburgh, Edinburgh EH8 9AB, UK; 2 IQANOVA Ltd., Edinburgh EH10 5LZ, UK; skyimager.uk@gmail.com (L.S.); goryanin@iqanova.org (I.V.G.); 3 geneXplain GmbH, 38300 Wolfenbüttel, Germany; alexander.kel@genexplain.com

**Keywords:** diagnostic metabolites, metabolomics, biomarkers, EHMN2026^®^, AI-QSP, quantitative systems pharmacology, genome-scale metabolic model, TRANSFAC^®^, TRANSPATH^®^, HumanPSD™, geneXplain, transcriptional regulatory potential, SBML, inborn errors of metabolism, oncometabolites

## Abstract

Background/Objectives: Diagnostic metabolites measured in newborn screening, inherited metabolic disease, lysosomal storage disease, oncometabolite testing and routine clinical biochemistry are direct read-outs of human metabolic state. Their mechanistic interpretation requires linking measured metabolites to enzymes, pathways, regulatory context, disease knowledge and, increasingly, AI-assisted quantitative systems pharmacology (AI-QSP) workflows. We developed EHMN2026^®^T as a license-aware AI-QSP integration framework that connects the EHMN2026^®^ metabolic backbone with licensed geneXplain knowledge resources while keeping ownership, licensing and redistribution constraints explicit. Methods: EHMN2026^®^T integrates the SBML-encoded EHMN2026^®^ metabolic backbone with licensed TRANSFAC^®^ 2025.2, TRANSPATH^®^ 2025.2 and HumanPSD™ 2025.2 resources. TRANSFAC^®^ position weight matrices were used for promoter-level analysis of EHMN metabolic genes. The resulting transcription factor (TF)–gene connections were mapped to EHMN genes, TRANSPATH^®^ signalling/molecular-state entries and HumanPSD™ disease/drug context. The framework is positioned as a controlled component of the IQANOVA AI-QSP environment, but only aggregate statistics, non-proprietary EHMN-derived summaries and manuscript-level examples are reported publicly unless separate permission is obtained from the relevant rightsholders. Results: Promoter analysis of 1681 EHMN2026^®^ metabolic genes using 1147 mapped TRANSFAC^®^ matrices identified 291,387 ENSG-level TF–gene regulatory-potential connections involving 398 TFs and 1,107,264 predicted binding sites. The diagnostic panel contained 80 covered genes (63.5%), including complete coverage of oncometabolite enzymes and high coverage of organic acidaemia, steroidogenesis and fatty-acid oxidation categories. Mapping to TRANSPATH^®^ expanded the EHMN genes into 144,529 molecular-state representations and 14,879 gene–pathway or gene–chain pairs. HumanPSD™ was used as a licensed translational context layer; EHMN-specific HumanPSD™ outputs are treated as license-controlled derived outputs and are therefore not redistributed as open detailed tables in this manuscript. Conclusions: EHMN2026^®^T provides a license-aware AI-QSP integration framework for tracing a diagnostic metabolite from a measured clinical value to candidate enzyme nodes, regulatory potential, signalling/molecular-state context and disease or therapeutic interpretation. PWM-derived TF–gene links are presented as regulatory hypotheses, not proof of active regulation. Public release should be limited to aggregate statistics and non-proprietary EHMN-derived components; detailed TRANSFAC^®^, TRANSPATH^®^ and HumanPSD™-derived edges, mappings, annotations and SBML outputs remain subject to geneXplain ownership and licensing terms.

## 1. Introduction

Diagnostic metabolites are among the most clinically actionable outputs of human metabolism. Newborn screening programs measure phenylalanine, acylcarnitines, organic acids, succinylacetone and other small molecules to detect inborn errors of metabolism. Specialist biochemical genetics laboratories quantify sphingolipids, glycogen-derived tetrasaccharides, oxysterols, porphyrin intermediates and related pathway products to support diagnosis and monitoring of lysosomal storage, carbohydrate, sterol and heme biosynthesis disorders. Oncology has added a further class of diagnostic metabolites, including 2-hydroxyglutarate in IDH1/IDH2-mutant malignancy, succinate in SDHx-related tumours and fumarate in fumarate hydratase deficiency [1,2,3,4,5,6,7,8,9,10].

These measurements are powerful because they are close to phenotypes. A metabolite concentration is an integrated read-out of enzyme activity, substrate supply, compartmental transport, cofactor availability, tissue state, inflammation, nutrition, drug exposure and therapy. The same feature also makes interpretation difficult. A raised metabolite may reflect a structural enzyme defect, altered transcriptional regulation, post-translational modification, pathway compensation, drug action or secondary disease context. A clinically useful computational resource should therefore connect the measured metabolite to the catalytic enzyme and then to the regulatory, pathway, disease and pharmacological context that can explain why the enzyme axis is altered [9,10,11,12,13,14,15,16,17,18,19,20].

Genome-scale metabolic reconstructions address the metabolic-network part of this problem. The Edinburgh Human Metabolic Network (EHMN) was one of the earliest community-scale reconstructions of human metabolism [21], and later independent reconstructions such as Recon, Recon3D and Human1 established genome-scale human metabolism as a practical framework for disease interpretation, pathway analysis and model-based hypothesis generation [22,23,24,25]. The recent EHMN2026^®^ reconstruction extends this foundation as a thermodynamically refined, SBML Level 3 Version 2/FBC2-compliant model with 22,642 reactions, 14,321 metabolite species and 3996 gene products [26,27,28]. It provides a curated metabolic backbone for systems pharmacology, disease modelling and biomarker interpretation. Like other genome-scale metabolic models, however, it is primarily stoichiometric. It describes what transformations can occur, but not which regulatory programs determine enzyme expression, molecular state or clinical actionability in a given context [12,13,14,15,16,17,27,28,29,30].

EHMN2026^®^T was developed to bridge this gap as an integration framework between public and proprietary databases [31,32,33,34,35,36,37,38,39] rather than an independently redistributable copy of proprietary knowledge bases. The framework links EHMN2026^®^ to three curated knowledge layers from geneXplain GmbH: TRANSFAC^®^ and TRANSCompel^®^ for transcription factor binding, promoter regulatory potential and composite regulatory-element context [31,32,33]; TRANSPATH^®^ for signalling, pathway steps, molecular states and regulatory pathway interpretation [37,38]; and HumanPSD™ for disease, drug, biomarker and clinical-trial context [39]. Previous applications of geneXplain-associated pathway walking and feedback-loop analysis illustrate how regulatory and pathway context can contribute to biomarker discovery, although detailed database-derived outputs remain license-controlled [40]. The EHMN2026^®^-derived metabolic backbone and any non-proprietary EHMN summaries remain under their respective ownership and licensing terms; TRANSFAC^®^, TRANSPATH^®^ and HumanPSD™ data, identifiers, annotations, mappings and derived detailed integration outputs remain subject to geneXplain ownership and licensing terms.

This work is also positioned within the emerging AI-QSP paradigm, in which artificial intelligence and machine-learning methods can support literature triage, model generation, parameter estimation, surrogate modelling, virtual population generation, evidence retrieval and model documentation while preserving mechanistic interpretability and expert oversight [41,42,43,44,45,46,47]. IQANOVA describes this translational deployment context as an AI-enabled model-informed drug-development and AI-QSP environment (www.iqanova.org accessed 1 January 2026) [48]. In the present manuscript, IQANOVA is referenced as a platform and workflow context for non-proprietary EHMN-derived analysis and for potential licensed-access deployment; this wording does not imply ownership or sublicensing rights over TRANSFAC^®^, TRANSPATH^®^ or HumanPSD™ content.

The objectives of this study were to: (i) describe the construction of a license-aware EHMN2026^®^T integration framework; (ii) quantify the aggregate regulatory and pathway coverage of metabolic genes and clinically used diagnostic-metabolite enzymes; (iii) illustrate how diagnostic metabolites can be interpreted mechanistically using selected examples; (iv) position the framework within a conservative AI-QSP workflow for hypothesis generation and mechanistic model extension; and (v) define publication, data availability and redistribution boundaries for proprietary geneXplain-derived layers.

## 2. Materials and Methods

### 2.1. Study Design and Integration Overview

This was a computational integration-framework and coverage-analysis study. EHMN2026^®^ was used as the metabolic backbone. Licensed geneXplain knowledge resources were integrated as regulatory, signalling/molecular-state and translational context layers. Integration was performed through gene identifiers, gene symbols, promoter-level PWM results, pathway/molecule identifiers and diagnostic-metabolite gene annotations. Public resources including HMDB, KEGG, Reactome, Rhea, UniProt, HGNC, MetaNetX and ChEBI were used as conceptual and identifier-mapping comparators where appropriate [9,10,11,12,13,14,15,16,17]. The analysis reports aggregate coverage statistics and selected examples suitable for publication without redistributing detailed proprietary database content.

### 2.2. Licensing, Ownership and Data Governance

EHMN2026^®^T consists of an EHMN2026^®^ metabolic backbone and an integration layer linking it to licensed geneXplain resources. EHMN2026^®^-derived components remain under the ownership and licensing terms of their respective owners. TRANSFAC^®^, TRANSPATH^®^ and HumanPSD™ data, identifiers, annotations, molecule states, pathway names, TF–gene mappings, disease/drug annotations and detailed derived integration outputs remain subject to geneXplain GmbH ownership and licensing terms and are not redistributed independently. Detailed edge lists, derived SBML files containing geneXplain-derived connections, molecular-state mappings or pathway/disease/drug annotations should be made available only to users who hold appropriate geneXplain licenses or under terms explicitly approved by geneXplain. IQANOVA-hosted EHMN2026^®^materials (www.iqanova.org) may describe the non-proprietary EHMN-derived backbone, aggregate statistics, workflow logic and platform context, but should not be used to sublicense or redistribute geneXplain-derived detailed content unless explicitly agreed with geneXplain. Supplementray Materials are available.

### 2.3. EHMN2026^®^ Metabolic Backbone

The metabolic layer was derived from EHMN2026^®^ [26], which extends the original EHMN reconstruction [21] with thermodynamic refinement, MetaNetX (MNXref) and ChEBI harmonization, Reactome pathway annotation and SBML Level 3 Version 2/FBC2 encoding [12,16,17,27,28]. EHMN2026^®^ contains 22,642 reactions, 14,321 metabolite species and 3996 gene products. In EHMN2026^®^T, this layer functions as the non-proprietary metabolic anchor for diagnostic-metabolite interpretation and for linking genes to license-controlled regulatory, signalling and translational context. The same backbone also supports AI-QSP use cases by providing a standards-compliant substrate for model extension, mechanistic hypothesis generation and downstream quantitative modelling [41,42,43,44,45,46,47].

### 2.4. TRANSFAC^®^ Transcriptional Regulatory Layer

TRANSFAC^®^ 2025.2 [31,32,33] provided the transcriptional regulatory layer. The curated TRANSFAC^®^ factor, site and matrix collections include transcription factor records, experimentally supported TF-DNA site evidence, position weight matrices (PWMs), promoter records, enhancer and silencer annotations, and ChIP-derived transcription factor–binding-site records. TRANSCompel^®^ provides additional composite regulatory-element context [32]. Open regulatory resources such as JASPAR and ENCODE, as well as independent catalogues of human transcription factors, provide useful comparators but were not redistributed as substitutes for the licensed TRANSFAC^®^ analysis [34,35,36]. In EHMN2026^®^T, TRANSFAC^®^-derived outputs are interpreted as promoter regulatory potential. They should not be treated as proof that a TF actively regulates a gene in a specific tissue, disease state or treatment condition without independent transcriptomic, chromatin or perturbation evidence.

A TF–gene connection was recorded when at least one site was predicted for at least one PWM mapped to that TF in the promoter of an EHMN gene. PWM-to-TF mapping was performed using the licensed TRANSFAC^®^ matrix annotation table. Of 1330 PWM columns in the input prediction matrix, 1147 (86.2%) carried a unique TF mapping and were used for TF-level aggregation. Public regulatory resources such as JASPAR and ENCODE provide useful conceptual comparators for interpreting TF-binding profiles and functional genomic evidence, but they were not used to redistribute licensed TRANSFAC^®^ content [28,29,30]. Detailed PWM-to-promoter, TF-to-gene and predicted-binding-site tables are treated as geneXplain-derived outputs and should not be redistributed publicly unless explicitly permitted by the applicable geneXplain license.

### 2.5. TRANSPATH^®^ Signalling and Molecular-State Layer

TRANSPATH^®^ 2025.2 [37,38] provided upstream signalling, pathway-step and molecular-state context. The database represents semantic reactions, pathway-step reactions, molecular-evidence reactions, molecules, genes, pathways and pathway chains. Importantly for diagnostic-metabolite interpretation, TRANSPATH^®^ distinguishes isoforms, complexes, post-translationally modified molecules and localization-specific forms. Public reaction and pathway resources such as Reactome and Rhea provide complementary non-proprietary pathway/reaction identifiers and were used as comparators for standards-aware interpretation [12,13]. In EHMN2026^®^T, TRANSPATH^®^-derived identifiers, pathway names, molecular states and reaction-level mappings are license-controlled integration outputs rather than independently redistributable EHMN data.

### 2.6. HumanPSD™ Disease, Drug and Biomarker Layer

HumanPSD™ 2025.2 [39] provided disease, drug, biomarker and clinical-trial translation. EHMN metabolic genes and TRANSFAC^®^-derived TFs were queried against HumanPSD™ to retrieve potential disease, drug–protein, biomarker and clinical-trial context. Public identifier systems and biomedical knowledge bases, including UniProt, HGNC, DrugBank and OMIM, provide complementary external identifiers and clinical-genetic context, but detailed EHMN-specific HumanPSD™ mappings remain license-controlled [14,15,19,20]. In this manuscript, HumanPSD™ is used as a licensed translational interpretation layer and is reported primarily through database-scale statistics and selected qualitative examples. Detailed EHMN-specific HumanPSD™ gene-disease, drug–protein, biomarker and clinical-trial mappings remain subject to geneXplain licensing and should be released only under approved license terms.

### 2.7. Diagnostic-Metabolite Gene Panel

A curated panel of 126 enzymes responsible for producing, consuming or transporting clinically used diagnostic metabolites was assembled from the newborn-screening, inborn-error, lysosomal storage disease, oncometabolite and metabolomics literature [1,2,3,4,5,6,7,8,9,10,19,20,49]. The panel covers aminoacidopathies, organic acidaemias, fatty-acid oxidation disorders, urea-cycle disorders, carbohydrate and glycogen disorders, lysosomal storage diseases, porphyrias, steroidogenic disorders, oncometabolite-linked cancers, lipid/cardiovascular disorders, purine/pyrimidine disorders and creatine/renal biomarkers. Panel genes were cross-mapped to the EHMN2026^®^ gene set by gene symbol and harmonized against public gene and protein identifiers where available [14,15].

### 2.8. Pipeline, Outputs, Validation and Redistribution Classes

The integration pipeline had four steps. First, gene–protein–reaction associations were parsed from the EHMN2026^®^ SBML file. Second, licensed TRANSFAC^®^ PWM promoter scans generated TF–gene regulatory-potential connections and binding-site counts. Third, licensed TRANSPATH^®^ tables expanded EHMN genes into molecule, isoform, complex, modified-state and pathway/chain contexts. Fourth, licensed HumanPSD™ tables were used to add disease, drug, biomarker and clinical-trial interpretation where available. Public identifier harmonization, where performed, used the same general principles applied in genome-scale modelling, including stable metabolite, reaction, gene, protein and pathway identifiers [9,10,11,12,13,14,15,16,17,27,48]. Outputs were classified into: (i) publishable aggregate statistics; (ii) non-proprietary EHMN-derived tables; (iii) manuscript-level illustrative examples; and (iv) license-controlled detailed integration outputs, including TF–gene edge lists, PWM mappings, TRANSPATH^®^ molecular-state/pathway mappings, HumanPSD™ disease/drug annotations and any SBML files containing those derived connections.

### 2.9. AI-QSP Platform Context and Use-Case Boundary

AI-QSP is used here to denote an AI-assisted quantitative systems pharmacology workflow in which mechanistic models, curated knowledge resources, literature-derived hypotheses, automated documentation and validation steps are combined under expert review. The AI-QSP/QSPaaS concept has recently been described as a strategy for improving model generation, parameter estimation, predictive modelling and scalable deployment in drug discovery and development [41,42,43,44,45,46,47]. In EHMN2026^®^T, this concept is implemented conservatively: AI-assisted interpretation may prioritize enzyme nodes, candidate regulatory programs, pathway context and model-extension hypotheses, but it does not replace biochemical validation, clinical judgement or licensing controls. The IQANOVA platform context (www.iqanova.org accessed 1 January 2026) [48] is therefore presented as a deployment route for non-proprietary EHMN-derived workflows and for controlled licensed-access analyses, not as a public redistribution channel for TRANSFAC^®^, TRANSPATH^®^ or HumanPSD™ content.

### 2.10. Online Access, User Protocol and Planned Tools

To address practical use and reproducibility, a user-facing protocol and online access route will be provided through IQANOVA resources at www.iqanova.org [48]. The protocol will describe how users can map a measured metabolite, gene, enzyme or diagnostic category to the EHMN2026^®^ metabolic backbone; inspect non-proprietary reaction, metabolite and gene–protein–reaction context; and, where licensed access is available, extend the interpretation to TRANSFAC^®^ regulatory-potential summaries, TRANSPATH^®^ signalling and molecular-state context and HumanPSD™ disease/drug/biomarker information. The protocol will distinguish between public examples, aggregate outputs and license-controlled detailed outputs.

Three user-facing tools are proposed. First, the EHMN2026^®^ Metabolic Network Explorer will support search of the non-proprietary metabolic backbone by metabolite, enzyme, gene, reaction, pathway, compartment and diagnostic biomarker, including SBML-compatible identifiers and public annotations where available. Second, the EHMN2026^®^T Diagnostic Metabolite Interpreter will guide users from a measured diagnostic metabolite to candidate enzyme nodes, EHMN reaction context, aggregate regulatory-potential summaries, pathway or molecular-state context and disease or therapeutic interpretation. Third, the AI-QSP Model Builder for Diagnostic Metabolism will support extraction of selected diagnostic-metabolite subnetworks as SBML-compatible model scaffolds for hypothesis generation, quantitative systems pharmacology and expert-reviewed AI-assisted model extension.

EHMN2026^®^ is intended to be maintained as a regularly updated resource. Updates may include corrected reaction annotations, additional metabolite and gene mappings, revised compartmentalization, improved thermodynamic constraints, new diagnostic-metabolite panels, additional public annotations and updated protocol examples. Versioned release notes will be used to distinguish the published EHMN2026^®^ backbone from later updates and from EHMN2026^®^T integration layers.

Access to future tools and new releases will require appropriate licensing. All new EHMN2026^®^/EHMN2026^®^T releases beyond the version described in this manuscript—including extended versions with additional expert curation, disease-specific modules, AI-QSP workflow support, commercial deployment, update services, APIs, hosted workflows or licensed geneXplain-derived integration layers—will be provided only under IQANOVA and/or geneXplain-approved institutional, subscription or commercial licensing terms. These tools and releases should not be interpreted as free public resources unless a specific release is explicitly designated as free in writing by the relevant rightsholder. This access model does not affect the licensing restrictions described above: detailed TRANSFAC^®^-, TRANSPATH^®^- and HumanPSD™-derived mappings, annotations, identifiers, pathway names, disease/drug links and SBML outputs remain subject to geneXplain-approved licensing terms and are not redistributed as unrestricted public data.

## 3. Results

### 3.1. EHMN2026^®^T Architecture

**Figure 1:**
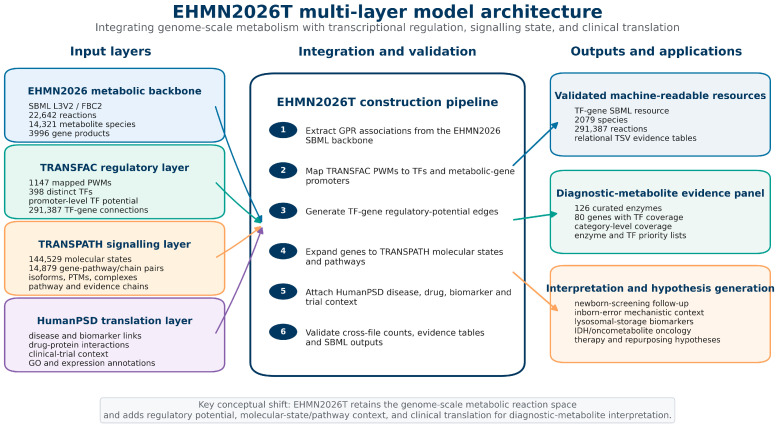
Architecture and data flow of EHMN2026^®^T. The EHMN2026^®^ metabolic reconstruction is used as the SBML Level 3 Version 2/FBC2 metabolic backbone and is linked to licensed geneXplain knowledge layers: TRANSFAC^®^ for promoter-encoded transcription factor regulatory potential, TRANSPATH^®^ for signalling, pathway and molecular-state context, and HumanPSD™ for disease, drug, biomarker and clinical-trial interpretation. The figure shows the conceptual framework and aggregate workflow. Detailed TRANSFAC^®^-, TRANSPATH^®^- and HumanPSD™-derived edges, identifiers, pathway names, annotations and SBML outputs remain subject to geneXplain ownership and licensing terms.

EHMN2026^®^T converts EHMN2026^®^ from a stoichiometric metabolic reconstruction into a license-aware AI-QSP integration framework for diagnostic-metabolite interpretation (Figure 1). The metabolic backbone anchors enzymes and gene–protein–reaction associations. The licensed TRANSFAC^®^ layer adds promoter-encoded transcriptional regulatory potential; the licensed TRANSPATH^®^ layer adds signalling and molecular-state context; and the licensed HumanPSD™ layer adds translational disease, drug, biomarker and clinical-trial context. The framework is therefore best understood as a controlled integration layer linking metabolic reactions to curated knowledge resources for expert-guided hypothesis generation, not as an open redistribution of those resources or as an autonomous diagnostic system.

### 3.2. Knowledge-Base Scale and EHMN-TRANSFAC^®^ Connectivity

The three licensed geneXplain knowledge resources linked to EHMN2026^®^T provide complementary evidence types and broad coverage of regulatory, signalling and translational biology (Table 1). TRANSFAC^®^ contributes promoter and TF-binding evidence, TRANSPATH^®^ provides signalling reactions and molecular-state expansion, and HumanPSD™ provides disease, drug and clinical-trial context. The scale of these resources also creates the main data-governance boundary: the manuscript can report aggregate statistics and derived interpretation principles, whereas detailed licensed edges, mappings, annotations and identifiers should remain access-controlled.

Promoter analysis of 1681 EHMN2026^®^ metabolic genes against the licensed TRANSFAC^®^ vertebrate matrix library produced 291,387 ENSG-level TF–gene regulatory-potential connections, corresponding to 291,109 unique gene-symbol–TF pairs and 1,107,264 predicted binding sites (Table 2). The mapped PWM set comprised 1147 matrices associated with 398 TFs. These values define the scale of the hypothesis space used for diagnostic interpretation. They should not be read as experimentally validated regulatory interactions and should not be redistributed as detailed edge lists without appropriate geneXplain permission.

### 3.3. TRANSPATH^®^ Molecular-State and Pathway Expansion

Mapping EHMN genes to licensed TRANSPATH^®^ content expanded 1681 metabolic genes into 144,529 distinct molecular-state representations (Table 3). Complex-type molecules comprised the largest category, indicating that many metabolic enzymes participate in multi-protein assemblies. Modified molecules represented an additional layer of post-translational context. These results are reported as aggregate counts; detailed molecule identifiers, pathway names, molecular states and reaction-level mappings remain subject to TRANSPATH^®^ licensing terms.

Pathway-level mapping connected 1310 EHMN gene symbols (78%) to at least one licensed TRANSPATH^®^ pathway or chain, yielding 14,879 unique gene–pathway or gene–chain pairs across 2331 pathway/chain entries (Table 4). The distribution was highly skewed, with a subset of hub genes connected to broad biological signalling or stress-response contexts. These mappings support interpretation of candidate upstream mechanisms but are not intended for open redistribution as detailed TRANSPATH^®^-derived tables.

### 3.4. Diagnostic-Metabolite Gene Coverage

**Figure 2:**
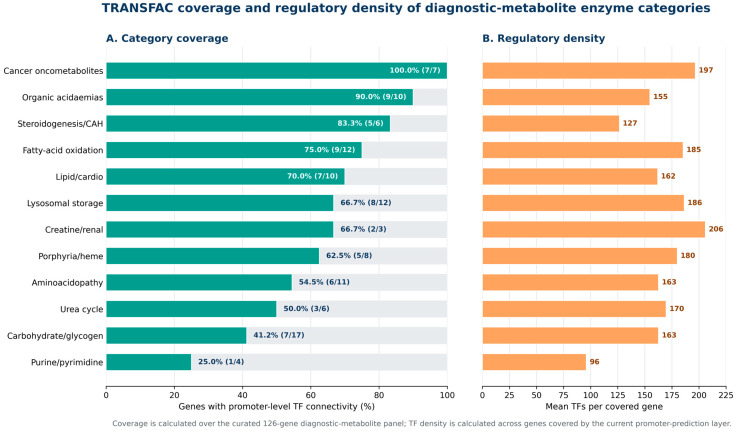
TRANSFAC^®^ regulatory-potential coverage of the diagnostic-metabolite gene panel by clinical category. Panel A shows the proportion of genes with promoter-level TF connectivity in each clinical category of the curated 126-gene diagnostic-metabolite panel. Panel B shows the mean number of distinct predicted TF regulators per covered gene. These are aggregate coverage statistics from a licensed TRANSFAC^®^-derived analysis and do not represent a public release of detailed TF–gene edge lists.

The curated diagnostic-metabolite panel comprised 126 enzymes. Of these, 80 (63.5%) were represented in the EHMN-TRANSFAC^®^-profiled gene set and therefore carried promoter-level TF regulatory-potential connectivity (Table 5; Figure 2). Coverage was complete for cancer oncometabolite enzymes (IDH1, IDH2, FH and SDHA-D), high for organic acidaemia, steroidogenesis and fatty-acid oxidation categories, and lower for aminoacidopathies and selected cofactor/vitamin pathways. The coverage analysis is reported at category and summary-gene levels to avoid redistribution of the full licensed TF–gene edge set.

The covered diagnostic-metabolite genes were not regulatorily sparse in the promoter-prediction layer. The mean number of distinct predicted TF regulators per covered diagnostic gene was 171.7, close to the EHMN-wide average of 173.5. Oncometabolite and creatine/renal categories showed particularly high regulatory-potential density. These observations are useful for prioritizing follow-up analyses, but they require independent validation using tissue-specific expression, chromatin accessibility, ChIP-seq or perturbation data before being interpreted as active regulatory programs.

### 3.5. Priority Enzyme and TF Nodes

**Figure 3:**
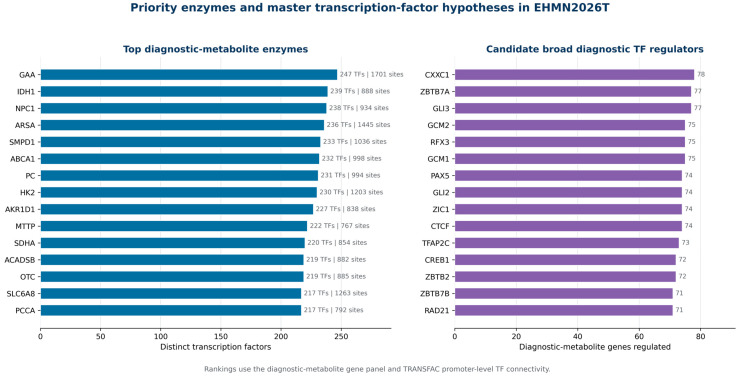
Prioritized diagnostic-metabolite enzymes and transcriptional regulators in EHMN2026^®^T. The left panel ranks the top diagnostic-metabolite enzymes by number of distinct predicted transcription factor regulators, with labels also reporting predicted promoter binding sites. The right panel ranks transcription factors by the number of diagnostic-metabolite genes for which promoter-level regulatory potential was detected. Rankings are intended for hypothesis generation and prioritization, not as proof of active regulation.

The most highly connected diagnostic-metabolite genes included clinically important disease enzymes (Table 6; Figure 3). GAA, the acid alpha-glucosidase gene mutated in Pompe disease and monitored using the urinary Glc4 biomarker, had 247 distinct predicted TF regulators and 1701 predicted promoter sites. IDH1, NPC1, ARSA, GBA1, SUCLG1, ABCC8, GALT and ASS1 were also highly connected. These rankings are best interpreted as prioritization scores for follow-up regulatory analysis rather than as validated disease-specific regulatory circuits.

At the regulator level, 391 of 398 profiled TFs (98%) were connected to at least one diagnostic-metabolite gene in the promoter-prediction layer. The top-ranked TFs included CXXC1, GLI3, ZBTB7A, GCM1, GCM2, RFX3, CTCF, GLI2, PAX5, ZIC1, TFAP2C, CREB1, RAD21 and NFKB1 (Table 7). This regulator set indicates that diagnostic-metabolite enzymes are embedded in a broad regulatory-potential landscape. The rankings do not establish tissue-specific regulation without orthogonal evidence.

### 3.6. Worked Examples

#### 3.6.1. Acid Sphingomyelinase Deficiency

ASMD is caused by biallelic SMPD1 defects and is associated with sphingomyelin accumulation and elevated lyso-sphingomyelin biomarkers. In EHMN2026^®^T, SMPD1 is connected to 233 TFs and 1036 predicted promoter sites. TRANSPATH^®^ provides licensed molecular-state and localization context for acid sphingomyelinase, while HumanPSD™ can be used under license to add disease, drug and clinical-trial context. This example illustrates how a measured sphingolipid biomarker can be connected to an enzyme node and candidate regulatory-potential context, while detailed proprietary annotations remain controlled.

#### 3.6.2. Pompe Disease

Pompe disease is caused by deficiency of acid alpha-glucosidase (GAA), with urinary glucose tetrasaccharide (Glc4) used as a non-invasive biomarker [38]. GAA was the most densely connected diagnostic-metabolite gene in this analysis, with 247 predicted TF regulators and 1701 predicted sites. EHMN2026^®^T places GAA in a lysosomal carbohydrate-metabolism context and provides a route to licensed disease/drug interpretation through HumanPSD™. The regulatory-potential ranking can prioritize tissue-specific validation, but it should not be interpreted as evidence of active transcriptional control in Pompe disease without additional data.

#### 3.6.3. IDH-Mutant Cancer

Somatic IDH1 and IDH2 mutations convert alpha-ketoglutarate to 2-hydroxyglutarate, creating an oncometabolite that is measurable by mass spectrometry and magnetic resonance spectroscopy [5,6]. EHMN2026^®^T captures IDH1 and IDH2 in the metabolic layer and adds promoter regulatory-potential context through licensed TRANSFAC^®^ outputs, while TRANSPATH^®^ and HumanPSD™ can provide signalling and therapeutic context under appropriate license conditions. The example demonstrates how oncometabolite interpretation can be extended from metabolite-enzyme mapping to candidate upstream and downstream hypotheses.

### 3.7. Comparison with Related Resources

EHMN2026^®^T differs from EHMN2026^®^, Recon3D and Human1 by linking a genome-scale metabolic backbone to licensed TF–gene, signalling/molecular-state and translational knowledge layers (Table 8). It differs from standalone regulatory or disease databases by retaining SBML-compatible metabolic anchoring. This comparison concerns analytical functionality and integration design; it does not imply that the geneXplain-derived layers are owned by IQANOVA or are independently redistributable as open EHMN content.

## 4. Discussion

### 4.1. Principal Findings

This study presents EHMN2026^®^T as a license-aware AI-QSP integration framework extending EHMN2026^®^ for diagnostic-metabolite interpretation. The principal finding is that a large proportion of clinically relevant diagnostic-metabolite enzymes can be connected, at aggregate level, to promoter-level TF hypotheses, molecular-state representations, pathway context and translational disease/drug information. The framework is therefore useful for mechanistic interpretation and QSP model-extension prioritization, provided that proprietary database content and derived detailed outputs remain governed by the applicable geneXplain licensing terms.

The scale of the EHMN-TRANSFAC layer is substantial: 291,387 TF–gene regulatory-potential connections across 1681 metabolic genes, involving 398 TFs and more than one million predicted binding sites. This should be read as a structured hypothesis space rather than a catalogue of experimentally active regulatory events. The size of the layer also reinforces the licensing issue: unrestricted release of detailed TF–gene edges or SBML encodings could expose licensed TRANSFAC^®^-derived predictions and mappings, and should therefore be avoided unless explicitly permitted.

### 4.2. Relevance to Metabolomics and Diagnostic Medicine

For metabolomics, EHMN2026^®^T provides a framework for moving from differential metabolite abundance to mechanistic interpretation. Instead of reporting only altered metabolites or enriched pathways, the framework can identify candidate enzyme nodes, regulatory-potential hypotheses and signalling contexts that may help formulate testable biological explanations. For AI-QSP, the same structure can support model-extension prompts, evidence retrieval, parameterization strategies, scenario generation and documentation workflows consistent with the AI-QSP/QSPaaS direction described by Goryanin, Goryanin and Demin [41] and with broader quantitative systems pharmacology and machine-learning approaches to drug development [34,35]. The intended use is decision support for mechanistic hypothesis generation, not autonomous clinical diagnosis or unvalidated AI-based interpretation.

The worked examples illustrate the general logic. In ASMD, sphingomyelin and lyso-sphingomyelin can be linked to SMPD1 and candidate regulatory-potential context. In Pompe disease, Glc4 can be linked to GAA and prioritized follow-up hypotheses. In IDH-mutant cancer, 2-hydroxyglutarate can be linked to IDH1/IDH2 and signalling or therapeutic context under license-controlled access. In each case, detailed proprietary annotations should be handled through licensed access rather than open redistribution.

### 4.3. Strengths and Novelty

The main strength of EHMN2026^®^T is the integration of four evidence layers that are usually analyzed separately: stoichiometric metabolism, promoter regulatory potential, signalling/molecular states and clinical translation. A second strength is that this integration can be used as an AI-QSP substrate for structured hypothesis generation, model-update prioritization and reproducible documentation while preserving expert oversight. A third strength is that the manuscript explicitly separates public aggregate analysis from license-controlled detailed outputs. This distinction makes the framework scientifically useful while reducing the risk of unintentionally redistributing proprietary TRANSFAC^®^, TRANSPATH^®^ or HumanPSD™ content.

### 4.4. Limitations

Several limitations should be considered. First, PWM-based promoter predictions identify regulatory potential, not necessarily active regulation in a specific tissue, cell type or disease state. Second, the present promoter scan focuses on proximal promoter regions and does not yet exploit the full enhancer, chromatin-accessibility or 3D-genome context. Third, HumanPSD™ is used here as a licensed translational context layer, but detailed EHMN-specific HumanPSD™ statistics are not yet reported and should not be overstated. Fourth, detailed TRANSFAC^®^, TRANSPATH^®^ and HumanPSD™-derived outputs may be derivative database products and cannot be assumed to be publicly redistributable. Fifth, diagnostic interpretation remains hypothesis-generating and requires validation against clinical, biochemical and omics data.

### 4.5. Future Development

Priority developments include completing promoter coverage for missing diagnostic genes such as PAH, ASS1, ASL, ARG1, MTHFR and FAH; integrating tissue-specific transcriptomics, chromatin accessibility and ChIP-seq evidence; adding enhancer-based regulatory evidence; performing EHMN-specific HumanPSD™ enrichment under appropriate license controls; and coupling selected high-priority diagnostic examples to validated quantitative models. A further development path is integration with the IQANOVA AI-QSP environment [48] for semi-automated model-extension proposals, evidence-linked documentation, scenario generation and expert-reviewed validation workflows. EHMN2026^®^ will be updated regularly, with versioned release notes distinguishing the published backbone from subsequent updated and extended releases. All new EHMN2026^®^/EHMN2026^®^T releases beyond the version described in this manuscript, as well as online tools, APIs, hosted workflows, expert-curated modules, update services and AI-QSP deployment services, will require appropriate licensing through IQANOVA and/or geneXplain-approved terms. Future public-facing releases should continue to separate any non-proprietary EHMN-derived summaries from license-controlled EHMN2026^®^T extensions and from TRANSFAC^®^, TRANSPATH^®^ and HumanPSD™-derived layers.

### 4.6. AI-QSP Deployment and Regulatory Boundary

The integration of AI with QSP is attractive because AI methods can accelerate literature triage, model generation, parameter exploration, surrogate modelling and virtual-patient workflows [34,35,39]. EHMN2026^®^T contributes a mechanistic metabolic and regulatory scaffold for this direction, but its use should remain evidence-linked and auditable. AI-generated hypotheses should be traceable to the metabolic backbone, licensed regulatory/pathway context, and validation evidence used to accept or reject a proposed model extension. From a governance perspective, IQANOVA-hosted AI-QSP deployment [48] should expose only non-proprietary summaries or licensed-access results according to the applicable contracts; it should not imply open public release of geneXplain-derived TF–gene edges, pathway names, molecular states, disease/drug annotations or SBML derivatives.

## 5. Conclusions

EHMN2026^®^T extends EHMN2026^®^ through a license-aware AI-QSP integration framework that links a human metabolic backbone with TRANSFAC^®^, TRANSPATH^®^ and HumanPSD™ knowledge layers. The framework identifies 291,387 TF–gene regulatory-potential connections across 1681 metabolic genes, expands metabolic genes into 144,529 molecular-state representations and covers 80 of 126 diagnostic-metabolite enzymes in the analyzed promoter layer. These results support mechanistic interpretation of diagnostic metabolites, prioritization of follow-up studies and expert-supervised AI-QSP model extension. EHMN2026^®^ is intended to be updated regularly; however, all new EHMN2026^®^/EHMN2026^®^T releases beyond the version described in this manuscript, including extended versions, support services, online tools and AI-QSP deployment tools, will require appropriate licensing through IQANOVA and/or geneXplain-approved terms. These resources should not be interpreted as free public releases unless explicitly stated in writing in the relevant release terms. These results do not establish active regulation without orthogonal evidence and do not imply public redistribution rights for licensed geneXplain-derived detailed outputs. A safe dissemination model is to publish aggregate statistics, non-proprietary EHMN-derived tables and workflow descriptions openly, while keeping TRANSFAC^®^, TRANSPATH^®^ and HumanPSD™-derived edges, annotations, identifiers, pathway names, disease/drug mappings and SBML representations accessible only under geneXplain-approved license terms. IQANOVA and www.iqanova.org may be cited as the AI-QSP platform context for non-proprietary EHMN-derived workflows and licensed-access deployment, but not as a sublicense or redistribution route for proprietary geneXplain resources.

## Figures and Tables

**Table 1:** Compositional statistics of licensed geneXplain knowledge resources linked to EHMN2026^®^ in the EHMN2026^®^T integration framework. Counts summarize source-resource scale and do not imply public redistribution rights for the underlying content.

Resource	Category	Count
TRANSFAC^®^ 2025.2	Transcription factor entries	50,937
TRANSFAC^®^ 2025.2	DNA sites	50,922
TRANSFAC^®^ 2025.2	Factor–DNA site links	68,898
TRANSFAC^®^ 2025.2	Position weight matrices (PWMs)	11,123
TRANSFAC^®^ 2025.2	Genes annotated	105,353
TRANSFAC^®^ 2025.2	Enhancers/silencers	231,413
TRANSFAC^®^ 2025.2	ChIP-derived TFBS entries	95,867,623
TRANSPro 2025.2	Human promoter sequences	89,839
TRANSCompel 2025.2	Composite regulatory elements	593
TRANSPATH^®^ 2025.2	Reactions (total)	1,253,744
TRANSPATH^®^ 2025.2	Semantic reactions	96,899
TRANSPATH^®^ 2025.2	Pathway-step reactions	19,734
TRANSPATH^®^ 2025.2	Molecular-evidence reactions	1,137,190
TRANSPATH^®^ 2025.2	Molecules	1,055,396
TRANSPATH^®^ 2025.2	Pathways	1595
TRANSPATH^®^ 2025.2	Pathway chains	1627
HumanPSD™ 2025.2	Human proteins	19,878
HumanPSD™ 2025.2	Diseases/disease models	2775
HumanPSD™ 2025.2	Drugs	9911
HumanPSD™ 2025.2	Gene–disease assignments	137,060
HumanPSD™ 2025.2	Drug–protein interactions	53,667
HumanPSD™ 2025.2	Disease annotations	414,870
HumanPSD™ 2025.2	Clinical trials	701,066
HumanPSD™ 2025.2	Clinical-trial–disease assignments	1,181,124
HumanPSD™ 2025.2	Clinical-trial–drug assignments	292,823

**Table 2:** Quantitative summary of the EHMN-TRANSFAC^®^ promoter connectivity layer.

Metric	Value	Interpretation
EHMN genes analyzed (ENSGs)	1681	Metabolic genes with mappable promoter
Unique gene symbols	1679	Two symbols collapse multiple ENSGs
PWM columns in input matrix	1330	Total predictive columns
PWMs with TF mapping	1147	Used for TF–gene attribution
PWMs without TF mapping	183	Excluded from final layer
Unique TFs profiled	398	TFs with ≥1 EHMN connection
TF–gene connections (ENSG-level)	291,387	Primary connectivity statistic
TF–gene pairs (symbol-level)	291,109	Symbol-collapsed view
Total predicted binding sites	1,107,264	Sum across all connections
Mean sites per TF–gene connection	3.80	Average site count
Mean TFs per gene	173.5	Promoter regulatory potential
Mean target genes per TF	732.1	TF reach across EHMN

Source files used internally for this analysis included EHMN_TRANSFAC_connectivity_statistics.tsv, EHMN_TRANSFAC_TF_statistics.tsv and EHMN_TRANSFAC_gene_statistics.tsv. Aggregate counts were cross-validated against an internal SBML representation of TF–gene regulatory-potential connections. The detailed TF–gene edge list and SBML representation may constitute geneXplain-derived outputs and are therefore not proposed for unrestricted public redistribution.

**Table 3:** TRANSPATH^®^ molecular-state expansion of EHMN2026^®^ metabolic genes.

Molecule Category	Count	Comment
EHMN metabolic genes (input)	1681	Mapped to TRANSPATH^®^
Total TRANSPATH^®^ molecule representations	144,529	~86× expansion
Complex-type molecules	133,170	Multi-protein assemblies
Modified molecules (_mod suffix)	9350	Post-translational states
Basic-type molecules	4801	Canonical protein entries
Isogroup-type molecules	3779	Isoform groups
Orthogroup-type molecules	2146	Cross-species ortholog groups
Family-type molecules	633	Protein family abstractions

**Table 4:** EHMN-TRANSPATH^®^ pathway and chain connectivity.

Statistic	Count
EHMN gene symbols mapped to ≥1 pathway/chain	1310
Unique gene–pathway/chain pairs	14,879
Unique TRANSPATH^®^ pathway/chain entries	2331
Unique pathway entries	1301
Unique chain entries	687
Unique metabolic-chain entries	225
Unique evidence-chain entries	118
Mean pathway/chain entries per annotated gene	11.35
Median pathway/chain entries per annotated gene	6
Maximum for one gene	370

**Table 5:** TRANSFAC^®^ regulatory coverage of the diagnostic-metabolite gene panel by clinical category.

Clinical Category	Genes Covered	Coverage	TF Edges	Tfs/Gene
Cancer oncometabolites (IDH1/2, FH, SDHA–D)	7/7	100%	1377	196.7
Inborn-error organic acidaemias	9/10	90%	1392	154.7
Steroidogenic/CAH enzymes	5/6	83%	633	126.6
Fatty-acid oxidation disorders	9/12	75%	1669	185.4
Lipid/cardiovascular disorders	7/10	70%	1133	161.9
Lysosomal storage diseases	8/12	67%	1491	186.4
Creatine deficiency/renal	2/3	67%	412	206.0
Porphyrias/heme synthesis	5/8	63%	900	180.0
Aminoacidopathies	6/11	55%	976	162.7
Urea-cycle disorders	3/6	50%	509	169.7
Carbohydrate/glycogen disorders	7/17	41%	1139	162.7
Purine/pyrimidine disorders	1/4	25%	96	96.0
TOTAL diagnostic-metabolite panel	80/126	63.5%	13,733	171.7

**Table 6:** Top 20 diagnostic-metabolite enzymes by TRANSFAC^®^ promoter regulatory connectivity.

Gene	Disease/Biomarker	TFs	Sites
GAA	Pompe disease; Glc4 tetrasaccharide	247	1701
IDH1	IDH-mutant cancer; 2-hydroxyglutarate	239	888
NPC1	Niemann–Pick C; cholestane-triol, oxysterols	238	934
ARSA	Metachromatic leukodystrophy; sulfatide	236	1445
SMPD1	ASMD/Niemann–Pick A,B; sphingomyelin, lyso-SM	233	1036
ABCA1	Tangier disease; HDL cholesterol	232	998
PC	Pyruvate carboxylase deficiency; lactate, alanine	231	994
HK2	Hexokinase 2 (glycolysis, Warburg)	230	1203
AKR1D1	Bile-acid synthesis defect; 3-oxo-Δ^4^ bile acids	227	838
MTTP	Abetalipoproteinemia; apoB lipoprotein	222	767
SDHA	Paraganglioma; succinate	220	854
ACADSB	SBCAD deficiency; C5 acylcarnitine isomer	219	882
OTC	OTC deficiency; orotic acid, ammonia	219	885
PCCA	Propionic acidaemia; propionic acid, C3	217	792
SLC6A8	Creatine transporter deficiency; creatine, creatinine	217	1263
PDHA1	PDH deficiency; lactate	216	896
INSR	Insulin signalling (diabetes)	215	999
ALAS1	Heme/porphyria axis; ALA	215	827
MDH2	TCA cycle (malate/fumarate)	214	899
SLC25A20	CACT deficiency; long-chain acylcarnitines	213	684

**Table 7:** Top transcription factors by number of diagnostic-metabolite-producing enzymes regulated in EHMN2026^®^T.

TF	Diagnostic Genes Regulated	TF	Diagnostic Genes Regulated
CXXC1	78	TFAP2C	73
GLI3	77	CREB1	72
ZBTB7A	77	ZBTB2	72
GCM1	75	RAD21	71
GCM2	75	ZBTB7B	71
RFX3	75	ZNF282	71
CTCF	74	ZNF449	71
GLI2	74	ZNF524	71
PAX5	74	IKZF1	70
ZIC1	74	PAX3	70
EGR2	69	ZNF443	70
EOMES	69	ZNF770	70
GLIS1	69	—	—

**Table 8:** Comparison of EHMN2026^®^T with related models and resources.

Feature	EHMN2026^®^T	EHMN2026^®^ [26]	Recon3D [24]	Human1 [25]
Genome-scale metabolism	Yes	Yes	Yes	Yes
Curated TF–gene regulatory layer	Yes (licensed TRANSFAC^®^ layer)	No	No	No
Promoter PWM analysis of metabolic genes	Yes (aggregate statistics; detailed edges controlled)	No	No	No
Signalling/molecular-state layer	Yes (licensed TRANSPATH^®^ layer)	No	No	No
Disease/drug/clinical-trial annotation	Yes (HumanPSD™)	Partial (Reactome)	Partial	Partial
SBML Level 3/FBC2 compliant	Yes	Yes	Yes	Yes
Reactome pathway annotation	Yes (via EHMN2026^®^)	Yes	Partial	Partial
Thermodynamic cycle elimination	Yes (via EHMN2026^®^)	Yes	No	Partial
Diagnostic-metabolite gene-panel coverage	63.5% (80/126)	Backbone only	Variable	Variable
Public redistribution of detailed proprietary-derived outputs	No (licensed access/geneXplain-approved terms only)	N/A	N/A	N/A

## Data Availability

Public data accompanying this manuscript should be restricted to aggregate statistics, non-proprietary EHMN-derived summaries, figure source data that do not expose licensed database content, user-protocol descriptions and workflow descriptions sufficient to understand the analysis. EHMN2026^®^T consists of an EHMN2026^®^ metabolic backbone and an integration layer linking it to licensed geneXplain resources. EHMN2026^®^-derived components remain under the ownership and licensing terms of their respective owners. EHMN2026^®^ is intended to be updated regularly. All new EHMN2026^®^/EHMN2026^®^T releases beyond the version described in this manuscript, including extended versions, online tools, APIs, hosted workflows, expert-curated modules, AI-QSP ^®^ deployment tools, update services and support services, will require appropriate IQANOVA and/or geneXplain-approved licensing and should not be interpreted as free public releases unless explicitly stated in writing in the applicable release terms. TRANSFAC^®^, TRANSPATH^®^ and HumanPSD™ data, annotations, identifiers, molecule states, pathway names, TF–gene links, disease/drug/biomarker annotations and detailed derived integration outputs remain subject to geneXplain GmbH ownership and licensing terms and are not redistributed independently. Detailed edge lists, mappings and SBML files containing geneXplain-derived content are available only to appropriately licensed users or under terms explicitly approved by geneXplain GmbH. No statement in this article should be interpreted as granting IQANOVA or any third party a right to sublicense or redistribute geneXplain proprietary resources.

## Supplementary Materials

The following supporting information can be downloaded at: https://www.mdpi.com/article/10.3390/metabo16070469/s1. The following supporting files will be supplied where they do not expose licensed geneXplain-derived content: Table S1, aggregate EHMN-TRANSFAC^®^ connectivity statistics; Table S2, aggregate TF statistics without detailed proprietary edge lists; Table S3, aggregate diagnostic-category coverage; Table S4, non-proprietary EHMN diagnostic-metabolite gene panel; Figure S1, workflow diagram; and a concise user protocol describing public EHMN2026^®^ exploration and license-controlled EHMN2026^®^T interpretation workflows. Supplementary Materials will not include future extended EHMN2026^®^/EHMN2026^®^T releases, online tools, APIs, hosted workflows, AI-QSP deployment services or licensed integration layers that require separate licensing. Detailed PWM-to-promoter mappings, TF–gene edge lists, TRANSPATH^®^ molecule/pathway mappings, HumanPSD™ disease/drug/clinical-trial annotations and SBML files containing geneXplain-derived connections should not be deposited as unrestricted public Supplementary Material unless explicitly permitted by geneXplain GmbH and the applicable license terms.
