# Supplementary material for: EHMN2026®T: A License-Aware AI-QSP Integration Framework Linking EHMN2026® with TRANSFAC®, TRANSPATH® and HumanPSD™ for Diagnostic-Metabolite Interpretation
Source: Metabolites. 2026 Jul 4;16(7):469. doi: 10.3390/metabo16070469 (PMC13413855; doi:10.3390/metabo16070469)
Supplement: Supplementary file 1 [file metabolites-16-00469-s001.zip › metabolites-4375080_Supplementary_Materials.pdf]

Supplementary Materials

Manuscript ID: metabolites-4375080

EHMN2026®T: A License-Aware AI-QSP Integration Framework Linking EHMN2026® with TRANSFAC®, TRANSPATH® and HumanPSD™ for Diagnostic Metabolite Interpretation

Supplementary data-governance note

These supplementary files provide aggregate statistics, non-proprietary EHMN-derived summaries, gene-level diagnostic-metabolite summaries and workflow figures. They intentionally do not include detailed PWM-to-promoter mappings, TF-gene edge lists, TRANSPATH® molecule/pathway mappings, HumanPSD™ disease/drug/clinical-trial annotations or SBML files containing geneXplain-derived connections. Such detailed outputs remain subject to the relevant geneXplain GmbH ownership and licensing terms.

Table S1. Aggregate EHMN-TRANSFAC® connectivity statistics

Aggregate summary of promoter-level regulatory-potential connectivity. This table reproduces summary counts only and does not disclose detailed TF-gene edge lists.

Metric	Value	Interpretation
EHMN genes analysed (ENSG)	1,681	Metabolic genes with mappable promoter
Unique gene symbols	1,679	Two symbols collapse multiple ENSG records
PWM columns in input matrix	1,330	Total predictive PWM columns in promoter analysis
PWMs with TF mapping	1,147	Mapped TRANSFAC® matrices used for TF-level attribution
PWMs without TF mapping	183	Excluded from final TF-level aggregation
Unique TFs profiled	398	Transcription factors with at least one EHMN connection
TF–gene connections (ENSG-level)	291,387	Primary aggregate regulatory-potential statistic
TF–gene pairs (symbol-level)	291,109	Gene-symbol-collapsed regulatory-potential view
Total predicted binding sites	1,107,264	Aggregate binding-site count across all TF–gene connections
Mean sites per TF–gene connection	3.80	Average number of sites per TF–gene connection
Mean TFs per gene	173.5	Mean promoter regulatory-potential density per EHMN gene
Mean target genes per TF	732.1	Mean EHMN gene reach per profiled TF
Diagnostic-metabolite genes covered	80 / 126 (63.5%)	Covered genes in the curated diagnostic-metabolite panel
Diagnostic TF edges	13,733	Aggregate TF–diagnostic-gene regulatory-potential count

Table S2. Aggregate TF statistics without detailed proprietary edge lists

Transcription factors ranked by the number of diagnostic-metabolite genes with predicted promoter-level regulatory potential. The full table is also provided in the accompanying spreadsheet and CSV file.

TF symbol	Diagnostic genes regulated
CXXC1	78
GLI3	77
ZBTB7A	77
GCM1	75
GCM2	75
RFX3	75
CTCF	74
GLI2	74
PAX5	74
ZIC1	74
TFAP2C	73
CREB1	72
ZBTB2	72
RAD21	71
ZBTB7B	71
ZNF282	71
ZNF449	71
ZNF524	71
IKZF1	70
PAX3	70
ZNF443	70
ZNF770	70
EGR2	69
EOMES	69

GLIS1	69
NFKB1	69
SALL1	69
SNAI2	69
ZNF532	69
ZNF616	69
GMEB2	68
ZBTB21	68
ZNF250	68
EBF1	67
GLI1	67
NFKB2	67
NR1I2	67
SRF	67
STAT3	67
TFAP2A	67
TFAP2B	67
THAP1	67
ZNF44	67
ZNF77	67
OSR1	66
RARA	66
ZBTB33	66
ZNF444	66
ZNF460	66
ZNF549	66
E2F4	65
E2F8	65
GTF2I	65
TP63	65
ZIC3	65
ZNF639	65
EGR3	64
HIF1A	64
ZNF554	64
ZNF669	64
ZNF697	64
GLIS2	63
NR1H4	63
RELA	63
SALL4	63
SMC3	63
TCF12	63
TFAP4	63
ZNF114	63
RUNX2	62
TFCP2	62
TP73	62
ZNF264	62
ZNF280D	62
ZNF701	62
MAFG	62
ARNT	61
KLF2	61
KLF8	61
RFX5	61
ZGPAT	61
ZNF658	61
ELF3	60
KLF1	60
PURB	60
ZBTB18	60
ZNF586	60
ZNF740	60
MAZ	59
NANOG	59
ZBTB14	59
PAX4	58
ZNF543	58
ZNF583	58
NFIC	58
XBP1	57
TFCP2L1	57
ZNF576	57
E2F1	56
RARB	56
ZNF140	56
LHX1	56
ZNF582	55
ZNF735	55
ZNF816	55
EGR1	54
ZIC4	54

NHLH1	54
KLF17	53
ZNF341	53
HEY2	52
RARG	52
VDR	52
ZNF331	52
NEUROD1	51
ZKSCAN3	51
ZNF322	51
ZNF563	51
ZSCAN29	51
E2F3	50
KLF12	50
SMAD5	50
ZNF121	50
ZNF836	50
HINFP	49
ZNF580	49
SALL2	48
UBP1	48
ZNF148	48
OLIG2	48
ZNF227	47
ZNF281	47
ZNF682	47
SMAD2	47
KLF3	46
NR2F6	46
PATZ1	46
ZNF23	46
BHLHE22	46
KLF4	45
MTF1	45
CREB3L1	44
SP2	44
SPDEF	44
ZNF286A	44
ZNF432	44
ZNF436	44
ETV6	44
KLF5	43
OVOL2	43
ZNF263	43
HNF1B	42
KLF15	42
PAX7	42
SP1	42
INSM1	41
ETV4	41
CREB3L4	40
FOXN1	40
RXRB	40
ZNF692	40
ETV1	40
EPAS1	39
ERG	39
GTF3C2	39
TFDP2	39
NFIB	39
TFAP2E	39
AHR	38
TFE3	38
ZNF219	38
HEY1	37
ZIC5	37
ZBTB24	37
CHURC1	36
NR2F1	36
GLIS3	36
HES7	35
CTCFL	35
ZNF841	35
PPARD	34
VEZF1	34
ZNF727	34
LIN28B	34
ETV7	34
MYCN	33
TFDP1	33
ZNF257	33
HES1	32
KLF10	32

RORB	32
MYOG	32
KLF13	31
LEF1	31
PBX3	31
ZBTB12	31
PBX1	30
SP3	30
ZFAT	30
ZNF574	30
NR5A2	30
RFX2	30
ZSCAN31	29
ELF1	29
KLF6	29
IKZF3	28
ZNF320	28
ZNF709	27
ZNF300	27
GABPA	27
HES2	26
SREBF1	26
ELF5	26
E2F2	26
HIC1	25
KLF11	25
FIGLA	25
ILF3	25
SP5	25
UNCX	25
CREB3	25
MAX	24
ZNF324	24
ZSCAN30	24
ZNF415	24
ZNF454	24
BMAL1	23
PLAGL2	23
TCF7	23
ZBTB49	23
ZNF30	23
ZNF37A	23
ZNF479	23
MAFA	23
E2F7	22
RUNX3	22
SFPQ	22
ETV3	21
MYC	21
SP4	21
FOXN2	21
TCFL5	21
PAX9	21
RERE	20
TCF7L1	20
ZNF778	20
EBF3	20
PAX6	20
ELF2	20
MLX	19
TP53	19
NR2C2	19
SP7	19
TAF1	19
FLI1	19
RXRG	18
ZNF823	18
NHLH2	18
KLF14	18
ZNF614	18
ATF6B	18
ATF3	18
SOHLH2	17
ZNF670	17
EGR4	17
ZBTB10	17
ZNF501	17
SP8	17
ELK1	17
HES5	16
ZNF232	16
ZNF213	16
DEAF1	16

ZNF3	16
OVOL1	16
ZNF142	15
TFEB	15
ZNF394	15
ZNF729	15
DIDO1	15
ZNF799	14
NFIX	14
PAX2	14
ZBTB37	14
GTF2IRD1	14
ZBED1	14
ZBTB6	14
ELF4	14
FEV	13
KLF9	13
ELK3	13
YY1	13
GABPB1	13
ZNF423	12
PLAG1	12
EHF	12
ZBTB5	12
ZNF343	12
ZNF679	11
ZBED2	11
FOXN4	11
CREM	11
ETS2	11
ZNF548	11
ZFP14	11
ARID4B	10
CLOCK	10
MNT	10
SATB2	10
USF2	10
ZBTB7C	10
ESR2	10
MAF	10
ETS1	10
FOXO3	10
ZNF383	10
ZNF610	10
PAX1	10
NRF1	10
YBX1	10
IRX5	10
E2F6	9
PKNOX1	9
ZNF684	9
ZNF557	9
ZNF672	9
KLF7	9
THAP7	9
YY2	9
ETV5	9
MGA	8
MLXIPL	8
RB1	8
ELK4	8
GMEB1	8
USF1	8
ZSCAN10	7
ATF1	7
NFYB	7
BHLHE40	7
MXI1	6
ZXDC	6
ZFX	6
NKX2-3	6
FOS	6
FOSB	6
JUN	6
JUNB	6
JUND	6
FOXL2	6
ZNF138	5
ATF2	5
ATF7	5
FOSL1	5
ERF	5
ETV2	5

ZFP57	4
ZNF76	4
ZNF594	4
CDX2	4
ZFP64	4
NFYA	4
PAX8	4
ASCL2	4
SP9	3
FOSL2	3
JDP2	3
ATF4	3
ZNF417	3
ZNF777	3
BHLHE41	3
CNOT3	3
ATF6	2
BATF3	2
CREB5	2
CARF	2
BCLAF1	2
ARNT2	2
IRX1	2
DMTF1	2
ZNF596	1
MLLT10	1
ZNF764	1
SETDB1	1
KLF16	1
ZNF329	1
NFYC	1
PROX1	1
RXRA	1
FOXR1	1
ZNF704	1

Table S3. Aggregate diagnostic-category coverage

Diagnostic-metabolite category coverage summarised at the category level. These are aggregate counts and do not contain detailed licensed edge lists.

Clinical category	Genes covered	Total genes	Coverage (%)	Total TF edges	Mean TFs per covered gene
Lysosomal storage disease	8	12	66.7	1491	186.4
Inborn error fatty acid oxidation	9	12	75.0	1669	185.4
Inborn error organic acids	9	10	90.0	1392	154.7
Urea cycle disorder	3	6	50.0	509	169.7
Aminoacidopathy	6	11	54.5	976	162.7
Porphyria / heme	5	8	62.5	900	180.0
Cancer oncometabolite	7	7	100.0	1377	196.7
Lipid / cardiovascular	7	10	70.0	1133	161.9
Carbohydrate / glycogen	7	17	41.2	1139	162.7
Steroidogenesis (CAH)	5	6	83.3	633	126.6
Purine / pyrimidine	1	4	25.0	96	96.0
Creatine / renal	2	3	66.7	412	206.0

Table S4. Non-proprietary diagnostic-metabolite gene panel: covered genes

Gene-level covered diagnostic-metabolite panel summary. The table lists gene identifiers, aggregate regulatory-potential counts and diagnostic role descriptions; it does not contain TF-gene edges, PWM mappings, proprietary pathway names or disease/drug annotation tables.

Gene symbol	Ensembl gene ID	Distinct predicted TF regulators	Predicted promoter binding sites	Mapped PWMs	Diagnostic role / biomarker context
GAA	ENSG00000171298	247	1701	529	Acid alpha-glucosidase (Pompe disease; glucose tetrasaccharide Glc4)
IDH1	ENSG00000138413	239	888	434	Isocitrate dehydrogenase 1 (2-HG oncometabolite in glioma/AML)
NPC1	ENSG00000141458	238	934	438	Niemann-Pick C1 (cholestane-triol,

					oxysterols)
ARSA	ENSG00000100299	236	1445	455	Arylsulfatase A (MLD; sulfatide)
SMPD1	ENSG00000166311	233	1036	433	Acid sphingomyelinase (ASMD/Niemann-Pick A/B; sphingomyelin, LysoSM)
ABCA1	ENSG00000165029	232	998	420	ABCA1 cholesterol efflux (Tangier disease)
PC	ENSG00000173599	231	994	468	Pyruvate carboxylase (lactate, alanine; PC deficiency)
HK2	ENSG00000159399	230	1203	456	Hexokinase 2
AKR1D1	ENSG00000122787	227	838	503	Delta-4-3-oxosteroid 5-beta reductase (bile acid)
MTTP	ENSG00000138823	222	767	423	MTP (abetalipoproteinemia)
SDHA	ENSG00000073578	220	854	394	Succinate DH A (paraganglioma; succinate)
ACADSB	ENSG00000196177	219	882	433	Short/branched-chain acyl-CoA DH
OTC	ENSG00000036473	219	885	436	Ornithine transcarbamylase (OTCD; orotic acid biomarker)
PCCA	ENSG00000175198	217	792	421	Propionyl-CoA carboxylase (PA; propionic acid, C3 acylcarnitine)
SLC6A8	ENSG00000130821	217	1263	395	Creatine transporter (CTD; creatine/creatinine)
PDHA1	ENSG00000131828	216	896	454	Pyruvate DH E1-alpha (lactate; PDH deficiency)
INSR	ENSG00000171105	215	999	428	Insulin receptor
ALAS1	ENSG00000023330	215	827	402	ALA synthase 1
MDH2	ENSG00000146701	214	899	449	Malate dehydrogenase 2
SLC25A20	ENSG00000178537	213	684	366	Carnitine-acylcarnitine translocase (CACT)
ABCG8	ENSG00000143921	209	837	377	Sterol efflux (sitosterolemia)
MMAB	ENSG00000139428	208	794	366	MMA cofactor assembly
ACO2	ENSG00000100412	208	916	410	Aconitase 2
IDH2	ENSG00000182054	207	805	362	Isocitrate dehydrogenase 2 (2-HG oncometabolite)
MTRR	ENSG00000124275	204	1121	381	Methionine synthase reductase
GLA	ENSG00000102393	201	847	355	Alpha-galactosidase A (Fabry; globotriaosylceramide Gb3, lyso-Gb3)
SDHB	ENSG00000117118	201	666	365	Succinate DH B (paraganglioma; succinate)
PDHB	ENSG00000168291	200	750	341	Pyruvate DH E1-beta
ETFDH	ENSG00000171503	199	699	354	Electron transfer flavoprotein DH (MADD/GA-II)
NAGS	ENSG00000161653	198	904	376	N-acetylglutamate synthase (urea cycle)
CPOX	ENSG00000080819	198	695	374	Coproporphyrinogen oxidase (HCP; coproporphyrins)
ACADS	ENSG00000122971	196	730	361	SCAD (short-chain acyl-CoA DH; C4 acylcarnitine)
CPT2	ENSG00000157184	196	721	452	Carnitine palmitoyltransferase 2 (CPT2; long-chain acylcarnitines)
PTS	ENSG00000150787	196	653	336	6-Pyruvoyltetrahydropterin synthase (BH4)
GATM	ENSG00000171766	195	690	324	Glycine amidinotransferase (creatine biosynthesis; creatinine)
HADHA	ENSG00000084754	194	745	332	LCHAD (long-chain 3-hydroxyacyl-CoA DH; C16-OH)
FECH	ENSG00000066926	194	660	337	Ferrochelatase (EPP; protoporphyrin IX)
BCKDHA	ENSG00000248098	191	803	354	Branched-chain amino

					acid catabolism (MSUD; leucine/isoleucine/valine)
BCKDHB	ENSG00000083123	189	585	342	Branched-chain amino acid catabolism (MSUD)
SDHD	ENSG00000204370	185	659	387	Succinate DH D (paraganglioma; succinate)
MMUT	ENSG00000146085	184	595	355	Methylmalonyl-CoA mutase (MMA; methylmalonic acid biomarker)
LCAT	ENSG00000213398	184	787	319	Lecithin-cholesterol acyltransferase (HDL)
ACADM	ENSG00000117054	180	511	302	MCAD (medium-chain acyl-CoA DH; C8 acylcarnitine, SIDS-linked)
SDHC	ENSG00000143252	178	583	309	Succinate DH C (paraganglioma; succinate)
TPO	ENSG00000115705	177	486	285	Thyroid peroxidase (hypothyroidism; T4)
ABCG5	ENSG00000138075	176	550	278	Sterol efflux (sitosterolemia; plant sterols)
CS	ENSG00000062485	175	598	328	Citrate synthase
HADHB	ENSG00000138029	166	498	279	Trifunctional protein beta
CBS	ENSG00000160200	166	579	278	Cystathionine beta-synthase (homocystinuria; homocysteine biomarker)
OGDH	ENSG00000105953	166	612	345	Oxoglutarate dehydrogenase
GCDH	ENSG00000105607	162	449	270	Glutaryl-CoA dehydrogenase (GA-I; glutaric acid, C5DC)
CYP21A2	ENSG00000231852	155	448	258	21-hydroxylase (classic CAH; 17-OHP biomarker)
PPOX	ENSG00000143224	154	442	254	Protoporphyrinogen oxidase (VP; protoporphyrins)
FH	ENSG00000091483	147	353	227	Fumarate hydratase (HLRCC; fumarate accumulation)
CYP11A1	ENSG00000140459	147	464	240	Side-chain cleavage
HEXB	ENSG00000049860	145	347	223	Beta-hexosaminidase B (Sandhoff)
PCCB	ENSG00000114054	144	357	222	Propionyl-CoA carboxylase (PA)
CYP27A1	ENSG00000135929	144	381	226	Sterol 27-hydroxylase (CTX; cholestanol biomarker)
UGT1A1	ENSG00000241635	143	369	225	UGT1A1 (Crigler-Najjar, Gilbert; bilirubin)
CYP11B1	ENSG00000160882	141	314	200	11-beta-hydroxylase (CAH)
SUOX	ENSG00000139531	141	363	208	Sulfite oxidase
ALAS2	ENSG00000158578	139	395	212	ALA synthase 2 (X-linked sideroblastic anemia)
MCCC1	ENSG00000078070	135	706	207	3-Methylcrotonyl-CoA carboxylase (3-MCC; C5-OH acylcarnitine)
IVD	ENSG00000128928	128	290	200	Isovaleryl-CoA dehydrogenase (IVA; C5 acylcarnitine biomarker)
HPD	ENSG00000158104	119	257	176	4-Hydroxyphenylpyruvate dioxygenase (tyrosinaemia III)
CYP17A1	ENSG00000148795	119	251	159	17-alpha-hydroxylase
G6PC1	ENSG00000131482	118	235	160	Glucose-6-phosphatase (GSD I; glucose, lactate)
HEXA	ENSG00000213614	115	308	238	Beta-hexosaminidase A (Tay-Sachs; GM2 ganglioside)
HMGCL	ENSG00000117305	114	534	201	HMG-CoA lyase (3-hydroxy-3-methylglutaric aciduria)
TAT	ENSG00000198650	107	219	140	Tyrosine aminotransferase (tyrosinaemia II)
CPT1A	ENSG00000110090	106	252	170	Carnitine palmitoyltransferase 1A

					(CPT1; C0/C16 ratio)
MCCC2	ENSG00000131844	100	225	141	3-Methylcrotonyl-CoA carboxylase (3-MCC)
XDH	ENSG00000158125	96	224	133	Xanthine dehydrogenase (xanthinuria; uric acid)
CPS1	ENSG00000021826	92	167	122	Carbamoyl phosphate synthase I (urea cycle)
HK1	ENSG00000156515	77	149	98	Hexokinase 1 (glucose phosphorylation)
GALC	ENSG00000054983	76	130	101	Galactosylceramidase (Krabbe; psychosine)
HSD3B2	ENSG00000203859	71	139	96	3-beta-HSD
ALDOB	ENSG00000136872	67	100	82	Aldolase B (hereditary fructose intolerance)
HMGCR	ENSG00000113161	62	115	86	HMG-CoA reductase (cholesterol; statin target)
CYP7A1	ENSG00000167910	48	67	54	Cholesterol 7-alpha-hydroxylase (bile acid synthesis)

Figure S1. Workflow diagram

Workflow for generating public, license-safe EHMN2026®T supplementary outputs from EHMN2026®, aggregate TRANSFAC® promoter-scan summaries, aggregate diagnostic-metabolite categories and non-proprietary gene-level summaries.

Figure S1. Workflow for generating public, license-safe EHMN2026®T supplementary outputs

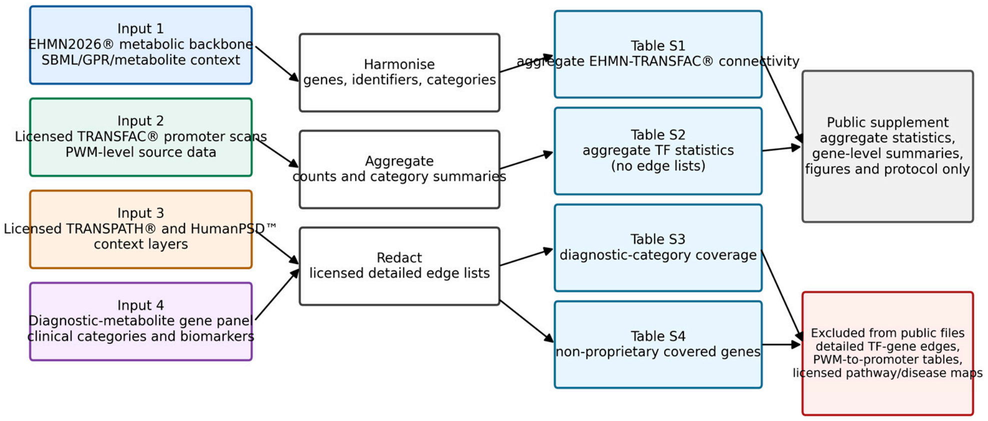

All public outputs are aggregate, category-level or gene-level summaries. No detailed proprietary geneXplain-derived edge lists or SBML derivatives are deposited as unrestricted supplementary files.

Figure S2. AI-QSP deployment boundary and licensing model

Conceptual deployment boundary separating public manuscript outputs, licensed EHMN2026®T/geneXplain-derived analyses and expert-reviewed AI-QSP workflows.

Figure S2. AI-QSP deployment boundary and licensing model for EHMN2026®T

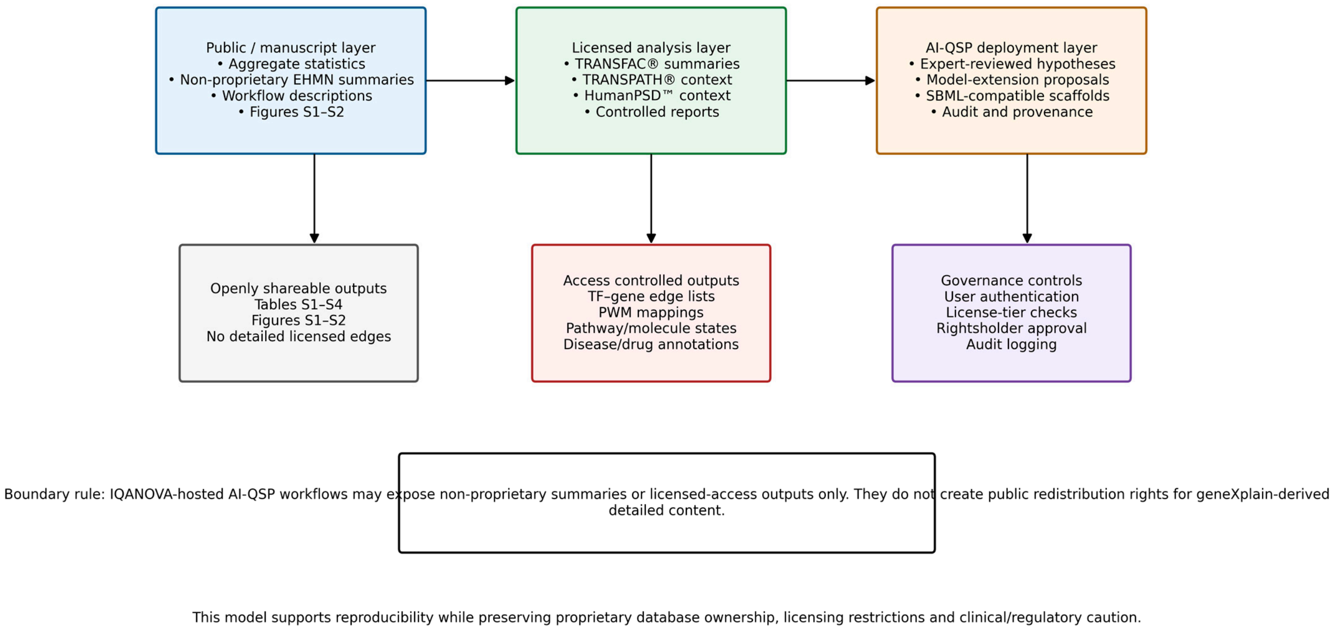

Included supplementary files

metabolites-4375080_Supplementary_Materials.docx — human-readable supplementary document

metabolites-4375080_Supplementary_Tables.xlsx — spreadsheet containing Tables S1–S4

Table_S1_aggregate_EHMN_TRANSFAC_connectivity_statistics.csv

Table_S2_aggregate_TF_statistics_no_edge_lists.csv

Table_S3_aggregate_diagnostic_category_coverage.csv

Table_S4_non_proprietary_diagnostic_metabolite_gene_panel_covered_genes.csv

Figure_S1_workflow_license_safe_supplement_generation.png / .svg

Figure_S2_AI_QSP_deployment_boundary_and_licensing_model.png / .svg
