# Supplementary figures and images for: EHMN2026®T: A License-Aware AI-QSP Integration Framework Linking EHMN2026® with TRANSFAC®, TRANSPATH® and HumanPSD™ for Diagnostic-Metabolite Interpretation
Source: Metabolites. 2026 Jul 4;16(7):469. doi: 10.3390/metabo16070469 (PMC13413855; doi:10.3390/metabo16070469)

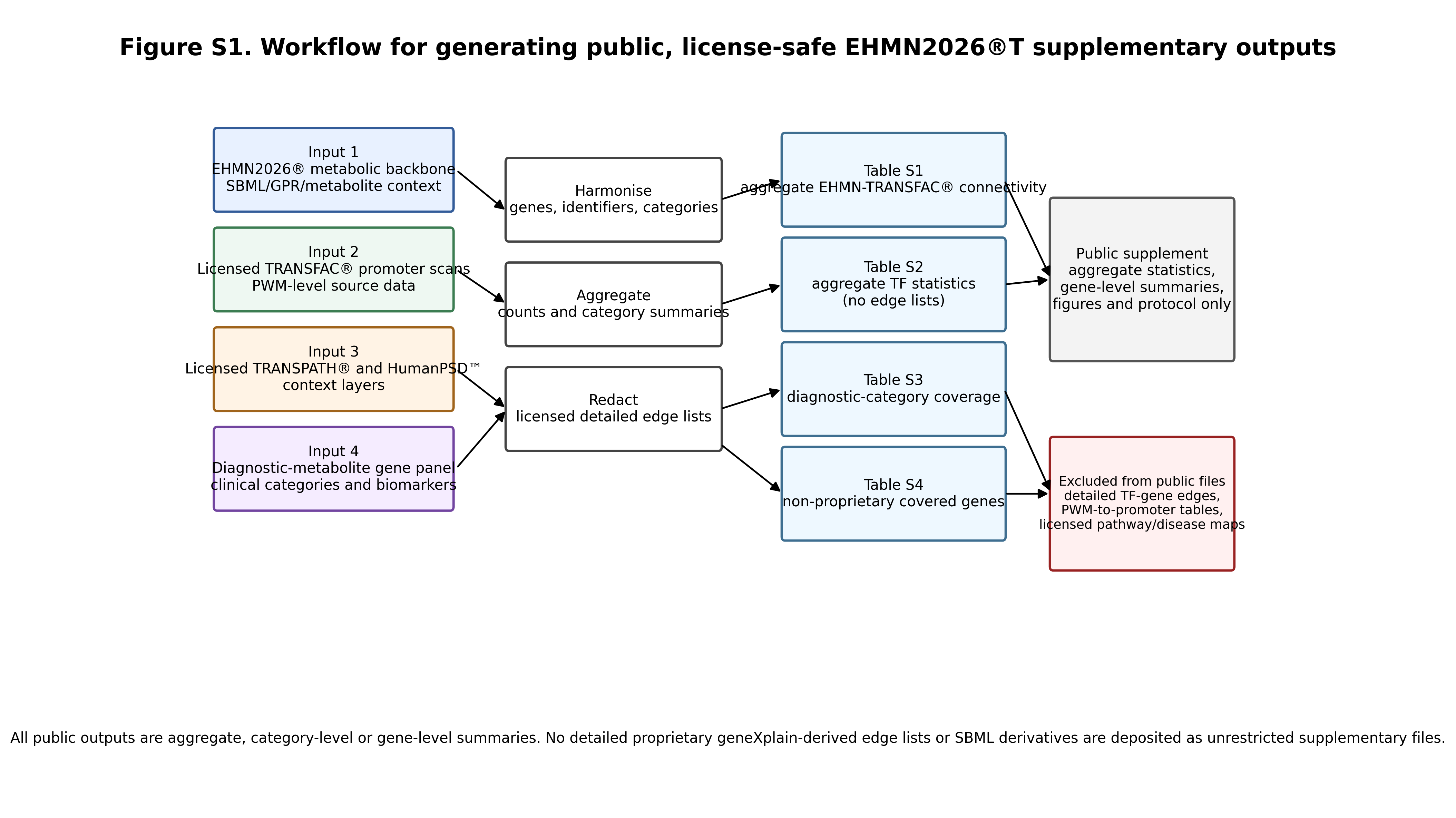

Supplement: Supplementary file 1 [file metabolites-16-00469-s001.zip › Figure_S1_workflow_license_safe_supplement_generation.png]

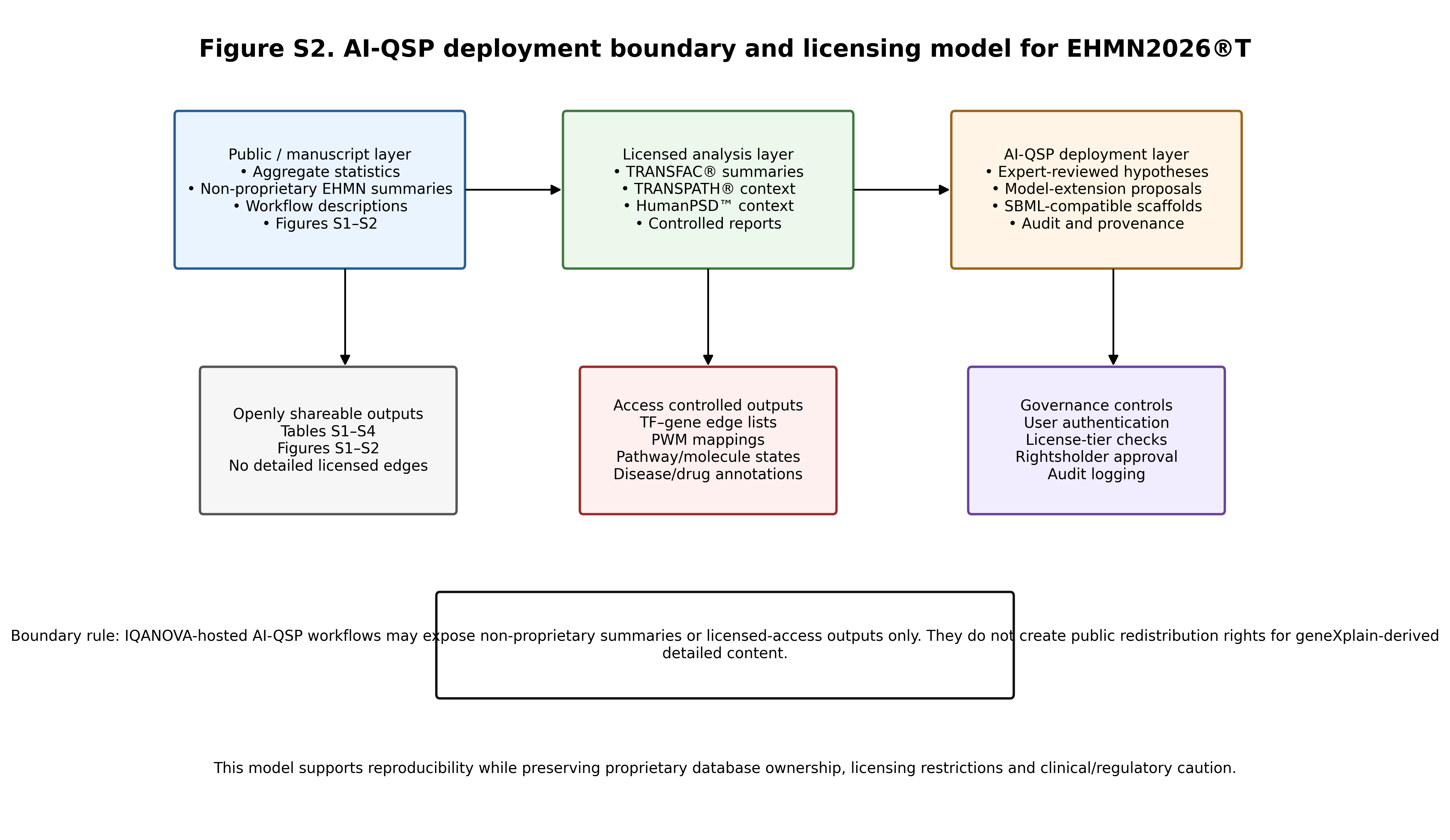

Supplement: Supplementary file 1 [file metabolites-16-00469-s001.zip › Figure_S2_AI_QSP_deployment_boundary_and_licensing_model.png]
